# Experience-dependent integration of a commercial AI system with C-TIRADS for thyroid nodule assessment: a retrospective single-center study

**DOI:** 10.3389/fendo.2026.1746242

**Published:** 2026-08-03

**Authors:** Jian Ding, Minjie Li, Yingxia Zhang

**Affiliations:** 1 Department of Ultrasound, Zhalantun People’s Hospital, Hulunbuir, China; 2 Department of Ultrasound, Affiliated Hospital of Inner Mongolia Medical University, Hohhot, China

**Keywords:** AI-assisted ultrasound, C-TIRADS, retrospective study, sonographer experience, thyroid nodules

## Abstract

**Objective:**

To evaluate the diagnostic performance of a commercial AI-assisted ultrasound system and C-TIRADS-based assessment among sonographers with different levels of experience, and to explore how two AI-assisted adjustment strategies performed in a retrospective clinical workflow for thyroid nodule evaluation.

**Methods:**

This retrospective single-center study included adult patients with thyroid nodules who underwent conventional ultrasonography and commercial AI-assisted analysis at our hospital between December 2022 and December 2023 and had available reference-standard outcomes. Three senior and three junior sonographers independently reviewed stored ultrasound images and videos and reached group-consensus C-TIRADS classifications while blinded to AI results and final diagnoses. Two AI-assisted integration strategies were evaluated: Strategy 1 required a one-level adjustment of the initial C-TIRADS category according to AI output, whereas Strategy 2 allowed flexible reconsideration after review of the AI result. Surgical histopathology or cytological results with follow-up-supported benign classification were used as reference standards. Diagnostic performance, agreement with reference standards, and biopsy-related indicators were compared across approaches.

**Results:**

A total of 389 thyroid nodules from 343 patients were included. Because the cohort required cytological or pathological confirmation and complete imaging data, the study population was enriched for malignancy (58.10%). The AI system showed higher sensitivity and accuracy than the junior sonographer group and comparable performance to the senior sonographer group. Among senior sonographers, the flexible strategy was associated with improvements in sensitivity, accuracy, negative predictive value, agreement, and AUC, whereas the mandatory strategy did not yield significant overall benefit. Among junior sonographers, the mandatory strategy was associated with improvements in sensitivity, accuracy, negative predictive value, agreement, and AUC, whereas the flexible strategy showed limited benefit. In this selected cohort, the more favorable strategy for each experience group was also associated with lower unnecessary biopsy rates and higher positive biopsy yields, without a significant increase in missed diagnoses.

**Conclusion:**

In this retrospective single-center, pathology-enriched cohort, the effect of AI-assisted C-TIRADS adjustment appeared to vary by sonographer experience. Mandatory adjustment may be more helpful for junior sonographers, whereas flexible integration may be more useful for senior sonographers. These findings should be interpreted as preliminary workflow observations and require validation in larger prospective multicenter studies.

## 1 Introduction

Thyroid nodules are a common clinical finding. While most are benign, a small proportion harbor clinically significant malignant potential (1). Globally, the incidence of thyroid cancer has risen consistently over recent decades, and this increase has been accompanied by growing concerns regarding overdiagnosis and overtreatment (2). In current practice, the diagnostic accuracy of thyroid ultrasound remains highly dependent on sonographer experience, due to the limited availability of robust quantitative and objective indicators. Significant inter-observer variability exists among examiners, particularly in the interpretation of suspicious ultrasound features and risk stratification of thyroid nodules (3, 4). These factors, together with inherent technical limitations such as ultrasound device resolution, contribute to considerable diagnostic variability. Consequently, unnecessary fine-needle aspiration biopsies or surgeries may occur, leading to patient harm and increased healthcare burdens. Thus, there is an urgent clinical need for novel methods and technologies to improve the accuracy and efficiency of thyroid nodule diagnosis and management, thereby facilitating personalized and precise care while mitigating overtreatment.

In response to the global refinement of Thyroid Imaging Reporting and Data Systems (TI-RADS), Chinese experts developed the “2020 Chinese Guidelines for Malignancy Risk Stratification of Thyroid Nodules by Ultrasound: C-TIRADS” to better align with domestic clinical practice. Compared with other commonly used systems such as ACR-TIRADS, C-TIRADS incorporates nodule size, number, and location into biopsy decision-making, while prior comparative studies have shown that different systems may achieve different trade-offs in specificity, simplicity, and unnecessary biopsy recommendations (5, 6). However, while such guidelines standardize examination procedures and image interpretation to some extent, subjectivity persists—particularly in assessing lesion characteristics such as size, morphology, and borders—due to variations in sonographer experience and perception. Integrating medical imaging data with artificial intelligence (AI) algorithms, particularly deep learning (DL), offers a promising approach to reducing this subjectivity. By training on large annotated ultrasound datasets, AI models can automatically detect, classify, and quantitatively assess lesions through pattern recognition and feature extraction (7, 8). This automated analysis helps minimize human factors in image interpretation, thereby improving the objectivity and consistency of diagnostic outcomes.

Currently, AI-assisted diagnostic systems based on thyroid ultrasound have been increasingly investigated as adjunctive tools for nodule assessment. Most existing studies have focused on static-image analysis or on direct comparisons between AI systems and human readers (9, 10). In the present study, we used a commercially available AI-assisted ultrasound system (ITS100) that provides real-time analysis during the scanning process. We did not develop, retrain, or modify this system. Instead, our aim was to evaluate how its output could be incorporated into routine C-TIRADS-based interpretation. Previous studies have suggested that this platform may provide useful adjunctive information for differentiating benign from malignant thyroid nodules (11, 12).

Despite growing interest in AI-assisted thyroid nodule evaluation, less is known about how existing AI tools should be integrated into clinical workflows for sonographers with different levels of experience, as prior studies have more commonly focused on comparisons between AI systems and readers of different experience levels rather than on workflow-level adjustment strategies (13). Therefore, the aim of this study was not to propose a new AI algorithm, but to assess, in a retrospective single-center setting, how two AI-assisted adjustment strategies—mandatory one-level adjustment and flexible reconsideration—performed when combined with C-TIRADS in junior and senior sonographer groups. In addition to diagnostic performance, we also examined biopsy-related indicators to provide a more practical assessment of workflow implications. We hypothesized that such an experience-aware human–AI interaction model may improve diagnostic consistency and reduce unnecessary biopsies by leveraging the complementary strengths of sonographer expertise and artificial intelligence. However, the present study was designed as an ultrasound-based workflow evaluation and was not specifically intended to assess whether AI improves discrimination in thyroid nodules with indeterminate cytology, such as Bethesda III/IV categories.

## 2 Materials and methods

### 2.1 Patient cohort and ethical approval

This retrospective single-center study enrolled adult patients with thyroid nodules who underwent thyroid ultrasound with concurrent AI-assisted analysis (ITS100) at the Affiliated Hospital of Inner Mongolia Medical University between December 2022 and December 2023 and had a definitive reference standard diagnosis (see below). The reference standard was defined as either surgical histopathology or fine-needle aspiration (FNA) cytology with confirmatory follow-up. Given the requirement for pathological verification and complete data, the study cohort constitutes a diagnostically enriched population, not a general screening sample. The study protocol was approved by the Medical Ethics Committee of the Affiliated Hospital of Inner Mongolia Medical University (Approval No. YJS2024128), and written informed consent was obtained from all participants. Preoperative FNA cytology data were used for case selection and, in a subset of benign nodules, as part of the reference-standard assessment together with follow-up stability (**Supplementary Table 1**). For nodules that underwent surgery, the final reference standard was surgical histopathology. Cytology itself was not used as an input variable for the AI system, and no dedicated subgroup analysis was performed for indeterminate cytology categories.

#### 2.1.1 Inclusion criteria

1. Age ≥18 years, with no gender restrictions;
2. Patients with thyroid nodules classified as C-TIRADS category 3 or higher, and with pathological results confirmed by ultrasound-guided FNA or surgery;
3. For cases with multiple nodules, those with available FNA or surgical pathology results were included;
4. FNA cytopathology classified as Bethesda II, IV, V, or VI, or Bethesda I/III with subsequent confirmation by surgical pathology;
5. Availability of complete clinical pathology records, ultrasound imaging data, and AI-assisted analysis data.

#### 2.1.2 Exclusion criteria

1. Patients with inconclusive or unsatisfactory FNA cytopathology results (Bethesda I or III) lacking surgical pathology confirmation;
2. Patients who had undergone partial or total thyroidectomy prior to enrollment;
3. Patients who received endocrine therapy, chemotherapy, radiotherapy, ablation, or radioactive iodine therapy;
4. Patients with incomplete clinical pathology records, ultrasound imaging data, or AI-assisted analysis data.

**Figure 1:**
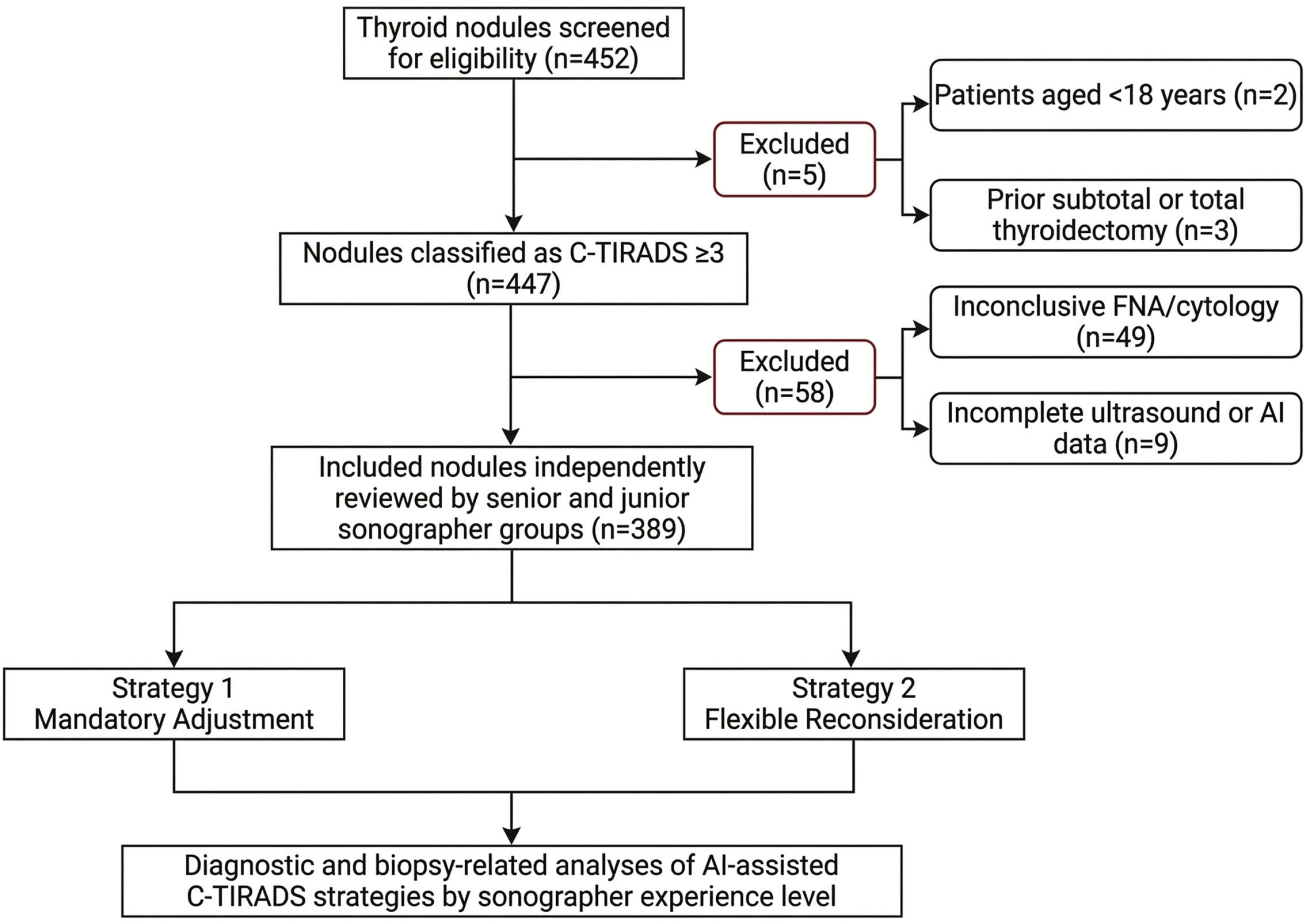
Flowchart of patient and nodule selection. Among 452 initially screened nodules, exclusions were applied according to predefined criteria, resulting in a final cohort of 389 thyroid nodules classified as C-TIRADS category 3 or higher and included in the comparative analysis.

The patient and nodule selection process is summarized in **Figure 1**.

### 2.2 Ultrasound examination and image analysis

Thyroid ultrasound examinations were performed using a GE LOGIQ S8 ultrasound system equipped with a GE L4-12tRS linear array probe (frequency range: 3–12 MHz). Each patient was examined in the supine position with the neck fully exposed. A senior ultrasound physician with more than 10 years of experience acquired all examinations using a standardized scanning protocol, including three sweeps of the thyroid gland in both transverse and longitudinal planes. Representative static images and dynamic videos were stored for subsequent review. The following sonographic features were documented for each nodule: location, size, orientation, margins, echogenicity, acoustic halo, echotexture, and the presence or absence of echogenic foci (14, 15).

For offline interpretation, three senior sonographers (each with more than 10 years of experience) and three junior sonographers (each with less than 5 years of experience) independently reviewed the stored ultrasound images and dynamic videos. The senior group and junior group each discussed cases internally and generated one consensus C-TIRADS classification per nodule (**Supplementary Table 2**). All six sonographers were blinded to the AI output and reference-standard diagnoses. Before case review, all participants underwent standardized training on the 2020 C-TIRADS guideline and the study-specific reading procedure (**Table 1**). According to guideline recommendations and previous evidence, C-TIRADS 4A or lower was considered negative and C-TIRADS 4B or higher was considered positive for binary performance analyses. This study therefore reflects group-level consensus performance rather than individual-reader variability (16).

**Table 1:** C-TIRADS categories and corresponding management recommendations used for binary analysis and biopsy-related evaluation.

Classification	Management recommendation
C-TIRADS 2	1. No FNA required; 2. It can be treated if oversized.
C-TIRADS 3	1. No FNA required; 2. It can be treated if oversized.
C-TIRADS 4A	1. >15 mm FNA; 2. Special nodule >10 mm FNA; 3. Single non-specific nodules ≤10mm may be monitored.
C-TIRADS 4B	1. >10mm FNA; 2. Special nodules >5mm FNA; 3. Special nodules <5mm: Consider comprehensive management based on individual circumstances; 4. Non-special nodules ≤10mm: Close follow-up may be indicated.
C-TIRADS 4C	Same as 4B
C-TIRADS 5	Same as 4B

Special nodules refer to those adjacent to the thyroid capsule, trachea, and recurrent laryngeal nerve, as well as multifocal nodules.

### 2.3 AI-assisted analysis

The Ian Thyroid Solution 100 (ITS100; MedPulse Intelligence Technology Co., Ltd.) is a commercially available proprietary computer-aided diagnosis system that was used as an adjunctive tool in this study. According to manufacturer information, the system analyzes time-series ultrasound video frames using convolutional neural-network-based image recognition and provides real-time localization and malignancy-risk output during scanning. The investigators did not develop, retrain, or modify the algorithm.

**Figure 2:**
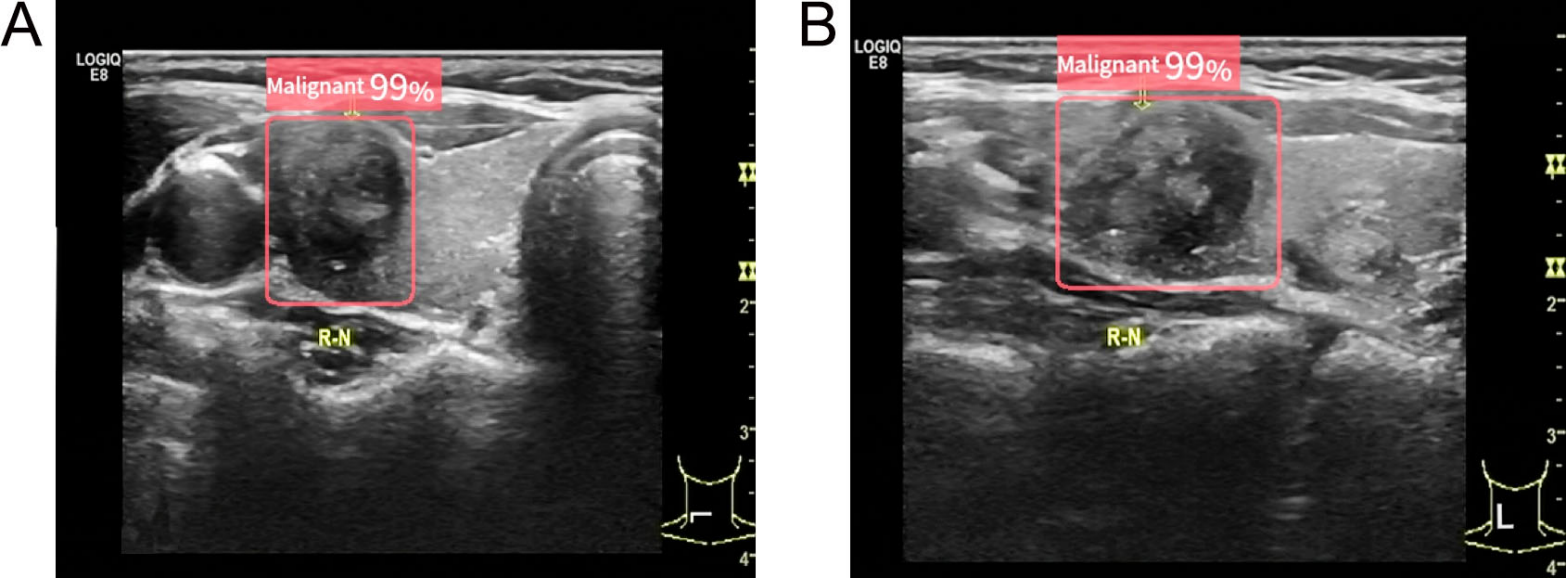
Representative case of concordant malignant AI output in both imaging planes. **(A)** Transverse-plane and **(B)** longitudinal-plane ultrasound images of a thyroid nodule in a 27-year-old woman show concordant high malignant-risk output (99%) on AI analysis. The final AI classification was “Possible Malignant.” Postoperative pathology confirmed papillary thyroid carcinoma.

**Figure 3:**
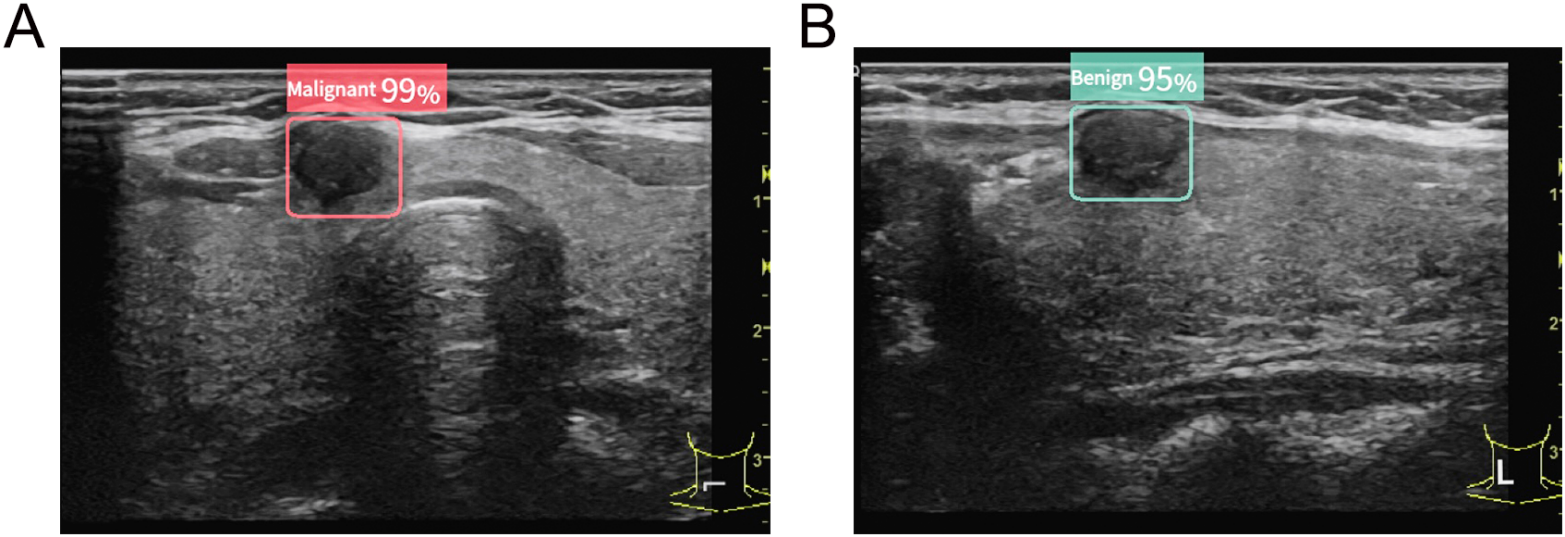
Representative case of discordant AI output between transverse and longitudinal planes. **(A)** Transverse-plane and **(B)** longitudinal-plane ultrasound images of a thyroid nodule in a 45-year-old man show a higher malignant-risk output on the transverse plane than on the longitudinal plane. According to the predefined rule, the more suspicious result was retained, and the final AI classification was “Possible Malignant.” Postoperative pathology confirmed papillary thyroid carcinoma.

**Figure 4:**
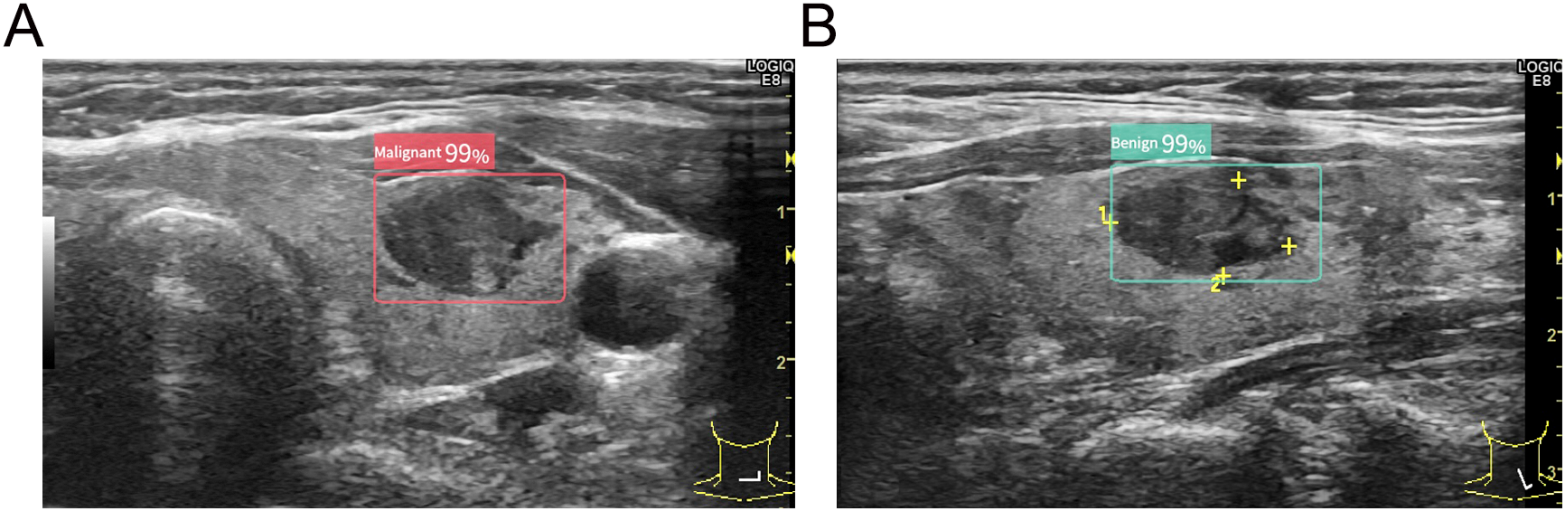
Representative case of equal malignant-risk output in both imaging planes. **(A)** Transverse-plane and **(B)** longitudinal-plane ultrasound images of a thyroid nodule in a 52-year-old woman show identical malignant-risk output (99%) on both planes. According to the predefined algorithm, the final AI classification was “Possible Malignant.” Postoperative pathology confirmed medullary thyroid carcinoma.

During the examination, the system displayed a probability output together with a color-coded interpretation: red for higher malignant risk and green for lower malignant risk (**Figure 2**). For each nodule, AI assessments were obtained from both transverse and longitudinal sweeps. When the two planes yielded different outputs, the more suspicious result was retained for the final AI classification used in this study (**Figure 3**). When the outputs were equivalent, the nodule was categorized as suspicious for malignancy (**Figure 4**). For binary analysis, “Possible Benign” was classified as negative and “Possible Malignant” as positive. A professional engineer supervised system operation to ensure standardized acquisition and data recording.

Because ITS100 is a commercial proprietary platform, full details of its internal training dataset, model parameters, and validation framework were not accessible to the investigators; this has been acknowledged as a limitation of the present study.

### 2.4 Combined diagnostic strategies

Two predefined AI-assisted integration strategies were evaluated after the baseline consensus C-TIRADS classification had been recorded for each nodule.

Strategy 1 (Mandatory Adjustment): if the AI result was “Possible Malignant,” the baseline C-TIRADS category was upgraded by one level; if the AI result was “Possible Benign,” the baseline category was downgraded by one level. C-TIRADS 5 was not upgraded further, and C-TIRADS 3 was not downgraded further.

Strategy 2 (Flexible Reconsideration): after reviewing the AI output together with the stored ultrasound images and dynamic videos, each sonographer group reconsidered the baseline C-TIRADS classification and decided by group consensus whether category adjustment was warranted.

**Figure 5:**
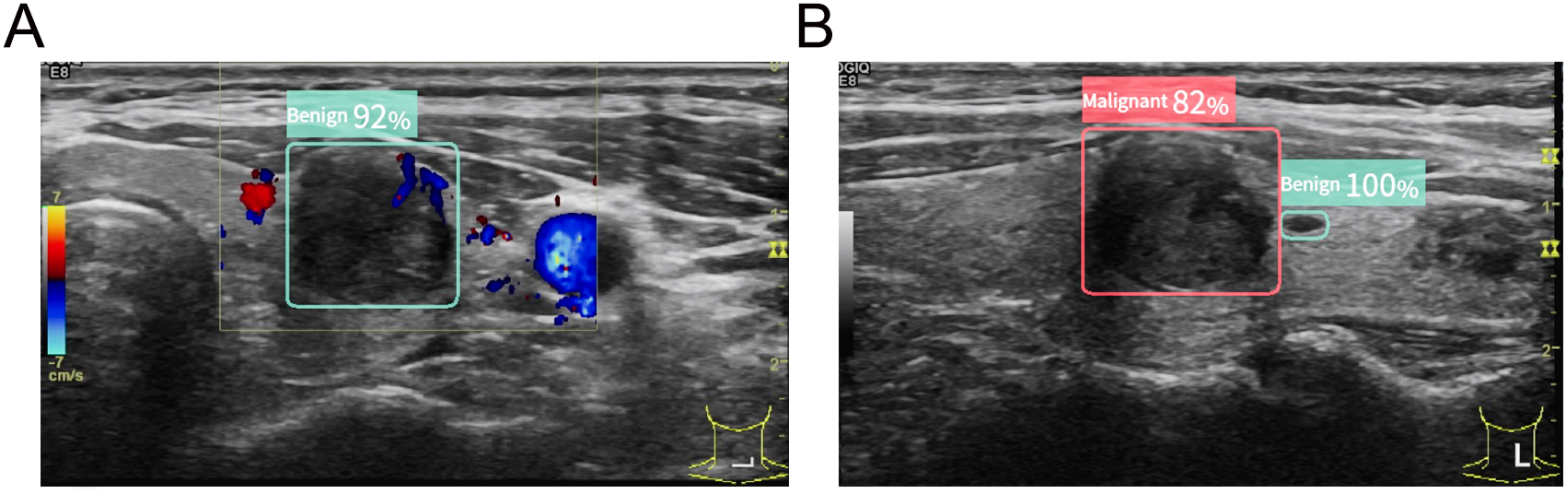
Representative case illustrating AI-assisted C-TIRADS adjustment. A thyroid nodule in a 32-year-old woman was located in the middle to lower third of the left thyroid lobe. **(A)** AI analysis suggested lower malignant risk on the transverse plane, whereas **(B)** the longitudinal-plane output remained less suspicious overall, yielding a final AI classification of “Possible Benign.” This case illustrates how AI output could influence mandatory or flexible C-TIRADS adjustment by sonographer groups.

The final category after each strategy was then used for binary diagnostic analysis and biopsy-related evaluation. A representative example illustrating AI-assisted C-TIRADS adjustment is shown in **Figure 5**.

### 2.5 Statistical analysis

Statistical analysis was performed using SPSS version 25.0. Continuous variables with normal or approximately normal distribution are presented as mean ± standard deviation and were compared using the independent-samples t test. Continuous variables that were not normally distributed are expressed as median (interquartile range) and were compared using the Mann–Whitney U test. Categorical variables are presented as numbers and percentages and were compared using the chi-square test or Fisher’s exact test, as appropriate.

Using the reference-standard outcomes as the gold standard, receiver operating characteristic (ROC) curves were plotted and the areas under the curves (AUCs) were compared using Z tests. Sensitivity, specificity, accuracy, negative predictive value (NPV), and positive predictive value (PPV) were calculated for each group. Agreement between each diagnostic approach and the reference standard was assessed using Cohen’s kappa. Unnecessary biopsy rate, positive biopsy rate, and missed diagnosis rate were calculated according to the C-TIRADS-based management criteria shown in **Table 1** and were compared before and after AI-assisted adjustment. All tests were two-sided, and P < 0.05 was considered statistically significant.

## 3 Results

### 3.1 Basic characteristics of nodules

A total of 343 patients with 389 thyroid nodules were included according to the predefined inclusion and exclusion criteria. Among the 343 patients, 82 were male (23.91%) and 261 were female (76.09%). Of the 389 nodules, 226 (58.10%) were malignant and 163 (41.90%) were benign. This relatively high malignancy prevalence likely reflects the selected nature of the study cohort, because only nodules with available reference-standard outcomes and complete imaging/AI data were included. The proportion of female patients with malignant nodules was significantly higher than that among patients with benign nodules (P < 0.05), and the mean diameter of malignant nodules was significantly smaller than that of benign nodules (P < 0.05) (**Table 2**).

**Table 2:** Baseline demographic, reference-standard, and ultrasonographic characteristics of the included thyroid nodules.

Variable	Final classification		*χ*^2^ /*t*/*Z*	*P*
	Benign	Malignant		
Gender [n (%)]			4.537	0.033
Male	46 (28.22)	43 (19.03)		
Female	117 (71.78)	183 (80.97)		
Age (years)	48.02±14.29	46.55±11.31	1.094	0.275
Nodule diameter (mm)	10.50 (7.20,16.50)	8.90 (6.20,12.48)	-3.275	0.001

Among the 226 malignant nodules, all were confirmed by surgical pathology. Of the 163 benign nodules, 24 were confirmed by surgical pathology, while the remaining 139 were classified as benign based on FNA results together with stable nodule size on at least two consecutive follow-up visits. Among the malignant nodules, 219 (96.90%) were papillary carcinomas, 2 (0.89%) were myxoid carcinomas, and 5 (2.21%) were follicular carcinomas. Among the benign nodules, 92 (56.44%) were nodular goiters, 29 (17.79%) showed inflammatory changes, 12 (7.36%) were follicular hyperplasia, and 30 (18.41%) were adenomas.

### 3.2 Analysis of C-TIRADS-related outcomes

#### 3.2.1 Diagnostic performance analysis by C-TIRADS group

All diagnostic indicators in the senior C-TIRADS group were significantly higher than those in the junior C-TIRADS group (P < 0.05 for all comparisons; **Table 3**). The senior C-TIRADS group demonstrated good agreement with reference standard, with a Kappa coefficient of 0.760 (indicating good agreement). In contrast, the junior C-TIRADS group showed moderate agreement, with a Kappa coefficient of 0.605.

**Table 3:** Diagnostic performance of baseline C-TIRADS assessment in the senior and junior sonographer groups.

	Senior baseline C-TIRADS	Junior baseline C-TIRADS	*χ*^2^/*Z*	*P*
Sensitivity(%)	86.28	78.76	4.433	0.035
Specificity(%)	90.80	82.82	4.527	0.033
Accuracy(%)	88.17	80.46	8.749	0.003
NPV(%)	82.68	73.77	4.212	0.040
PPV(%)	92.86	86.41	4.667	0.031
AUC	0.885	0.808	-4.920	0.000

#### 3.2.2 Univariate analysis of ultrasound features for thyroid nodules

Univariate analysis of ultrasound characteristics based on the C-TIRADS guidelines revealed statistically significant differences between benign and malignant thyroid nodules in terms of composition, echogenicity, margins, echogenic foci, orientation, echotexture, halo, and location (all P < 0.05; **Table 4**).

**Table 4:** Univariate analysis of C-TIRADS-related ultrasound features in benign and malignant thyroid nodules.

Variable		Final classification		*χ* ^2^	*P*
		Malignant [n(%)]	Benign [n (%) ]		
Composition				11.836	0.005*
	Solid	216(60.67)	140(39.33)		
	Predominately solid	7(36.84)	12(63.16)		
	Predominately cystic	2(25.00)	6(75.00)		
	Cystic and Spongiform	1(16.67)	5(83.33)		
Echogenicity				339.422	<0.001*
	Hyperechoic	0(0.00)	2(100.00)		
	Isoechoic	22(31.43)	48(68.57)		
	Hypoechoic	13(11.11)	104(88.89)		
	Markedly hypoechoic	191(99.48)	1(0.52)		
	Anechoic	0(0.00)	8(100.00)		
Margin				118.470	<0.001*
	Circumscribed	50(28.74)	124(71.26)		
Irregular margin	158(80.20)	39(19.80)		
Ill-defined	7(100.00)	0(0.00)		
Extrathyroidal extension	11(100.00)	0(0.00)		
Echogenic foci				14.241	0.004*
	Comet-tail artifacts	0(0.00)	2(100.00)		
Punctate echogenic foci of undetermined significance	115(64.61)	63(35.39)		
Macrocalcifications	19(38.78)	30(61.22)		
Peripheral calcifications	3(37.50)	5(62.50)		
No echogenic foci	89(58.55)	63(41.45)		
Orientation				66.605	<0.001
	Vertical	153(78.46)	42(21.54)		
	Horizontal	73(37.63)	121(62.37)		
Echotexture				128.305	<0.001
	Homogeneous	53(28.49)	133(71.51)		
	Heterogeneous	173(85.22)	30(14.78)		
Halo				83.420	<0.001
	Absent halo	102(47.66)	112(52.34)		
Even a thick halo	1(3.85)	25(96.15)		
Even a thin halo	31(68.89)	14(31.11)		
Uneven thin halo	27(81.82)	6(18.18)		
Uneven thick halo	65(91.55)	6(8.45)		
Location				81.847	<0.001
	Isthmus	12(46.15)	14(53.85)		
Upper	162(79.41)	42(20.59)		
Lower	27(34.18)	52(65.82)		
Middle	25(31.25)	55(68.75)		

### 3.3 Analysis of commercial AI-assisted results

#### 3.3.1 Diagnostic performance of the commercial AI system

The AI-assisted group demonstrated significantly higher sensitivity and accuracy compared to the C-TIRADS low-experience group (P < 0.05 for both metrics), whereas no statistically significant differences were observed in these metrics when compared to the C-TIRADS high-experience group (**Table 5**). The agreement between the AI-assisted group and pathological findings, indicated by a Kappa coefficient of 0.711, was intermediate between that of the junior sonographers (Kappa = 0.605) and the senior sonographers (Kappa = 0.760).

**Table 5:** Diagnostic performance of the commercial AI system compared with C-TIRADS-based assessment by senior and junior sonographer groups.

Group	Sensitivity(%)	Specificity(%)	Accuracy(%)	NPV (%)	PPV (%)	AUC
Senior C-TIRADS	86.28	90.80	88.17	82.68	92.86	0.885
AI-assisted analysis	86.73	84.66	85.86	82.14	88.69	0.857
*χ*^2^/*Z*	0.019	2.850	0.922	0.017	2.223	-1.097
*P*	0.891	0.091	0.337	0.895	0.136	0.272
Junior C-TIRADS	78.76	82.82	80.46	73.77	86.41	0.808
AI-assisted analysis	86.73	84.66	85.86	82.14	88.69	0.857
*χ*^2^/*Z*	5.020	0.203	4.048	3.552	0.510	1.713
*P*	0.025	0.652	0.044	0.059	0.475	0.087

#### 3.3.2 Frequency and impact of inconsistent AI predictions between transverse and longitudinal sections

**Figure 6:**
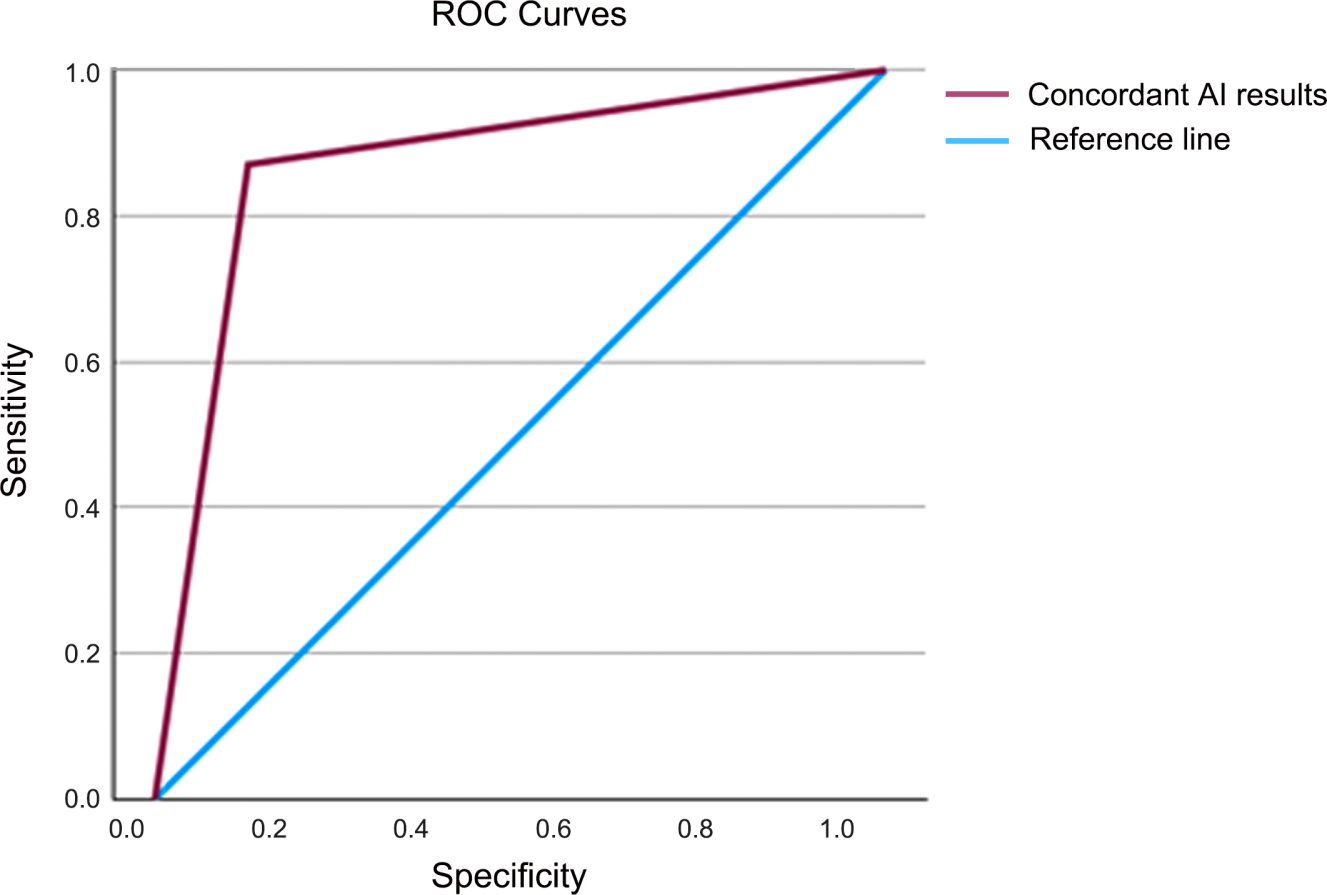
Receiver operating characteristic (ROC) curve for nodules with concordant AI results between transverse and longitudinal planes.

**Figure 7:**
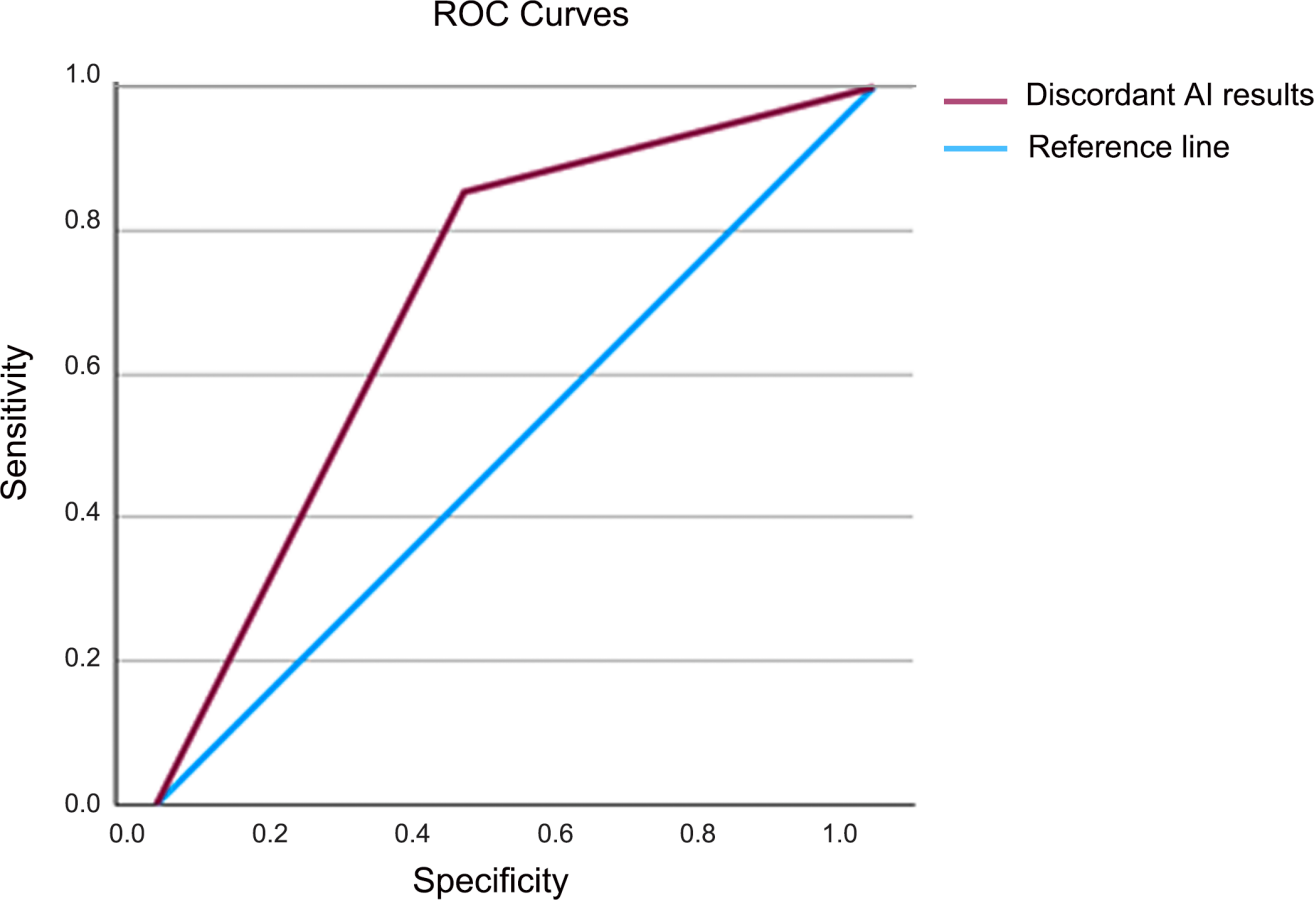
Receiver operating characteristic (ROC) curve for nodules with discordant AI results between transverse and longitudinal planes.

Among the 389 nodules evaluated, the commercial AI system produced inconsistent predictions between transverse and longitudinal section analyses in 55 cases (14.14%), whereas consistent results were obtained in 334 cases (85.86%). Diagnostic performance metrics were calculated separately for these two groups. The analysis revealed that the consistent group demonstrated significantly higher specificity and negative predictive value (NPV) than the inconsistent group (P < 0.05, **Table 6**). Furthermore, the area under the receiver operating characteristic (ROC) curve was greater for the consistent group (AUC = 0.871) than for the inconsistent group (AUC = 0.713) (**Figures 6** and **7**). Univariate analysis of baseline characteristics and ultrasound features indicated statistically significant differences in nodule diameter and location between the two groups. Specifically, a nodule diameter of <10 mm and an isthmus location were identified as factors associated with inconsistent AI predictions (**Supplementary Table 3**).

**Table 6:** Diagnostic performance of nodules with concordant versus discordant AI results between transverse and longitudinal planes.

	Concordant AI predictions	Discordant AI predictions	*χ* ^2^	*P*
Sensitivity(%)	87.03	85.37	0.080	0.777
Specificity(%)	87.25	57.14	8.933	0.003
Accuracy(%)	87.13	78.18	3.112	0.078
NPV(%)	84.42	57.14	6.508	0.011
PPV(%)	89.44	85.37	0.554	0.457
AUC	0.871	0.713		

#### 3.3.3 Analysis of factors affecting AI-assisted diagnostic performance

Univariate analysis of patient information and nodule ultrasound characteristics for patients correctly and incorrectly diagnosed by commercial AI system revealed statistically significant differences between the correctly diagnosed group and the misdiagnosed group in terms of age, halo, and location (**Table 7**).

**Table 7:** Factors associated with correct versus incorrect AI classification.

Variable		Diagnosis [n(%)]	Misdiagnosis [n(%)]	*χ* ^2^	*P*
Gender				2.341	0.126
	Female	262(87.33)	38(12.67)		
	Male	72(80.90)	17(19.10)		
Age (years)				4.146	0.042
	<55	228(83.52)	45(16.48)		
	≥55	106(91.38)	10(8.62)		
Echogenicity				3.078	0.515*
	Hyperechoic	2(100.00)	0(0.00)		
	Isoechoic	58(82.86)	12(17.14)		
	Hypoechoic	97(82.91)	20(17.09)		
	Markedly hypoechoic	169(88.02)	23(11.98)		
	Anechoic	8(100.00)	0(0.00)		
Orientation				0.560	0.454
	Vertical	164(84.54)	30(15.46)		
	Horizontal	170(87.18)	25(12.82)		
Echogenic foci				5.976	0.165*
	Comet-tail artifacts	1(50.00)	1(50.00)		
	Punctate echogenic foci of undetermined significance	158(88.76)	20(11.24)		
	Macrocalcifications	43(87.76)	6(12.24)		
	Peripheral calcifications	6(75.00)	2(25.00)		
	No echogenic foci	126(82.89)	26(17.11)		
Margin				0.837	0.848*
	Circumscribed	148(85.06)	26(14.94)		
	Irregular margin	171(86.80)	26(13.20)		
	Ill-defined	6(85.71)	1(14.29)		
	Extrathyroidal extension	9(81.82)	2(18.18)		
Nodule diameter				1.687	0.194
	<10mm	175(83.73)	34(16.27)		
	≥ 10mm	159(88.33)	21(11.67)		
Echotexture				0.042	0.838
	Homogeneous	159(85.48)	27(14.52)		
	Heterogeneous	175(86.21)	28(13.79)		
Halo				13.225	0.010*
	Absent halo	173(80.84)	41(19.16)		
	Even a thick halo	26(100.00)	0(0.00)		
	Even a thin halo	41(91.11)	4(8.89)		
	Uneven thin halo	32(96.97)	1(3.03)		
	Uneven thick halo	62(87.32)	9(12.68)		
Location				11.156	0.011
	Isthmus	19(73.08)	7(26.92)		
	Upper	172(84.31)	32(15.69)		
	Lower	76(96.20)	3(3.80)		
	Middle	67(83.75)	13(16.25)		

*Calculated using Fisher’s exact probability method.

### 3.4 Evaluation of AI-assisted C-TIRADS strategies

#### 3.4.1 Diagnostic efficacy of the integrated AI-assisted C-TIRADS strategy

**Figure 8:**
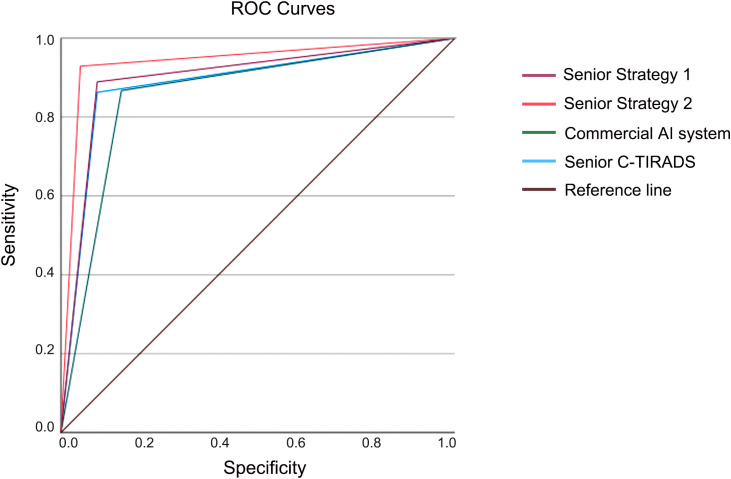
Receiver operating characteristic (ROC) curves for the senior sonographer group before and after AI-assisted adjustment. Baseline C-TIRADS performance is compared with Strategy 1 (mandatory adjustment) and Strategy 2 (flexible reconsideration).

After Strategy 1, no statistically significant change was observed in the main diagnostic indicators of the senior group, although the kappa coefficient increased from 0.760 to 0.791. After Strategy 2, the senior group showed significant improvements in sensitivity, accuracy, negative predictive value, and AUC, with the kappa coefficient increasing from 0.760 to 0.874 (all P < 0.05; **Figure 8** and **Table 8**).

**Table 8:** Diagnostic performance of AI-assisted C-TIRADS strategies in senior and junior sonographer groups.

Group	Sensitivity(%)	Specificity(%)	Accuracy (%)	NPV (%)	PPV (%)	AUC Value
Senior C-TIRADS	86.28	90.80	88.17	82.68	92.86	0.885
Senior Strategy 1	88.94	90.80	89.72	85.55	93.06	0.899
*χ*^2^/*Z*	0.734	0.000	0.471	0.541	0.006	-0.637
*P*	0.392	1.000	0.493	0.462	0.936	0.524
Senior C-TIRADS	86.28	90.80	88.17	82.68	92.86	0.885
Senior Strategy 2	92.92	95.09	93.83	90.64	96.33	0.940
*χ*^2^/*Z*	5.343	2.292	7.598	4.769	2.537	-3.074
*P*	0.021	0.130	0.006	0.029	0.111	0.002
Junior C-TIRADS	78.76	82.82	80.46	73.77	86.41	0.808
Junior Strategy 1	91.59	87.12	89.72	88.20	90.79	0.894
*χ*^2^/*Z*	14.737	1.177	13.130	11.369	2.075	-3.817
*P*	<0.001	0.278	<0.001	<0.001	0.150	0.000
Junior C-TIRADS	78.76	82.82	80.46	73.77	86.41	0.808
Junior Strategy 2	81.42	89.57	84.83	77.66	91.54	0.855
*χ*^2^/*Z*	0.499	3.119	2.590	0.762	2.727	1.713
*P*	0.480	0.077	0.108	0.382	0.099	0.087

**Figure 9:**
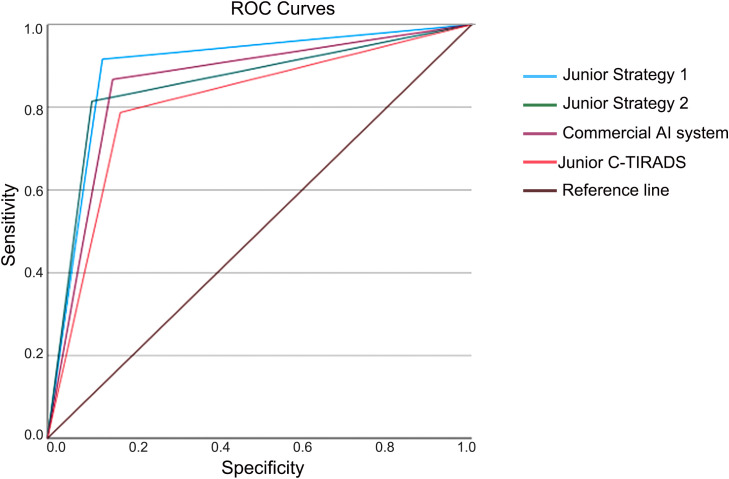
Receiver operating characteristic (ROC) curves for the junior sonographer group before and after AI-assisted adjustment. Baseline C-TIRADS performance is compared with Strategy 1 (mandatory adjustment) and Strategy 2 (flexible reconsideration).

In the junior group, Strategy 1 was associated with significant improvements in sensitivity, accuracy, negative predictive value, and AUC, and the kappa coefficient increased from 0.605 to 0.788 (all P < 0.05; **Figure 9** and **Table 8**). By contrast, Strategy 2 showed no statistically significant improvement in the primary diagnostic metrics, although the kappa coefficient increased to 0.695.

**Figure 10:**
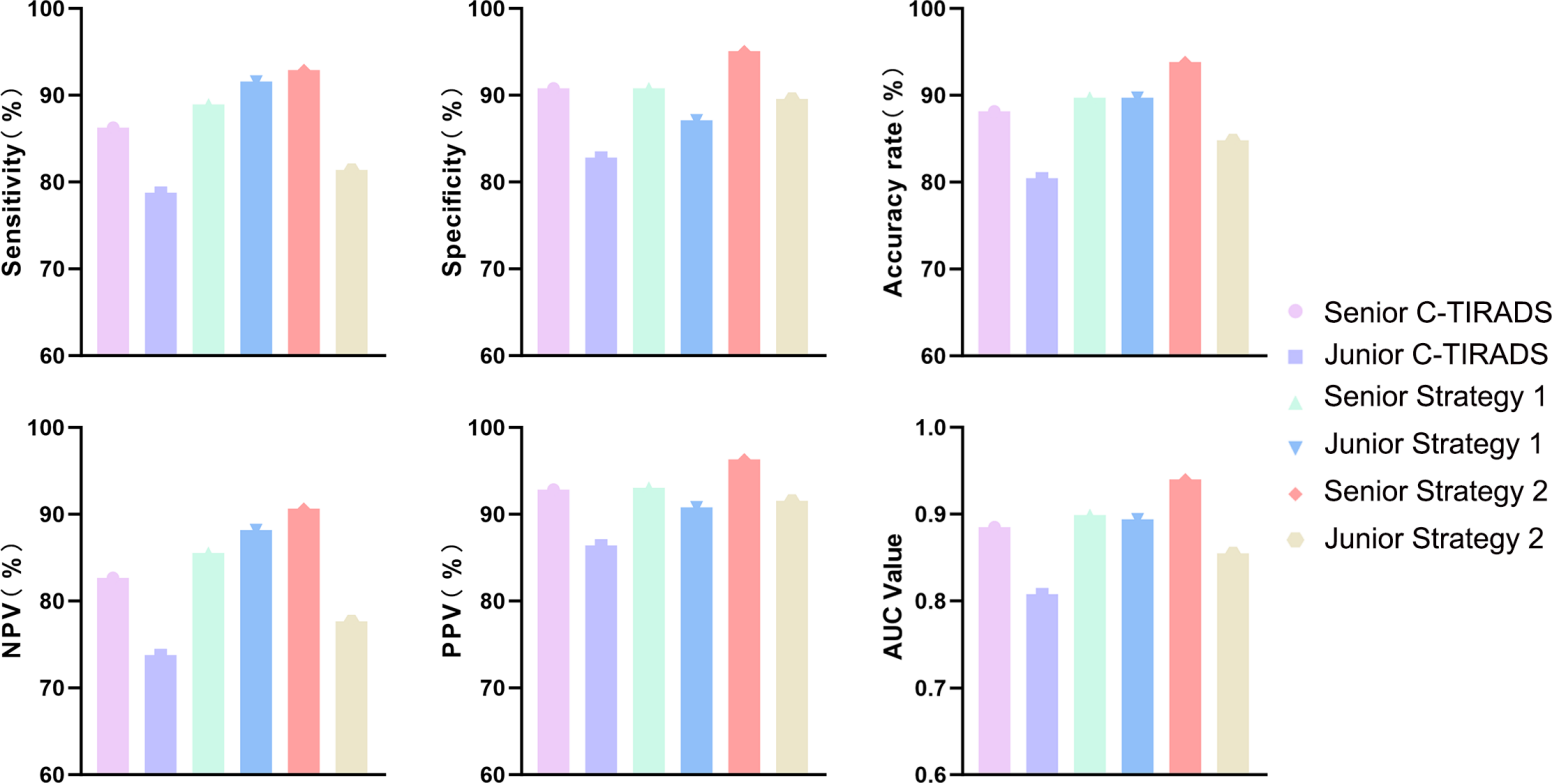
Comparison of diagnostic performance across AI-assisted strategies in senior and junior sonographer groups. Performance metrics are shown for baseline C-TIRADS, Strategy 1 (mandatory adjustment), and Strategy 2 (flexible reconsideration).

Notably, the performance of the junior group after Strategy 1 was comparable to that of the senior baseline group across the evaluated metrics (**Figure 10**). Overall, these findings suggest that the effect of AI-assisted adjustment differed between experience groups in this cohort.

#### 3.4.2 Analysis of the effect of AI-assisted C-TIRADS adjustment on biopsy-related indicators

In the senior group, both Strategy 1 and Strategy 2 were associated with lower unnecessary biopsy rates and higher positive biopsy rates, without a statistically significant increase in the missed diagnosis rate (**Table 9**).

**Table 9:** Biopsy-related indicators before and after AI-assisted C-TIRADS adjustment in senior and junior sonographer groups.

Group	FNA Count (n)	Malignant nodules in FNA(n)	Benign nodules in FNA (n)	Unnecessary biopsy rate (%)	Positive rate (%)	Missed Diagnosis Rate (%)
Senior C-TIRADS	136	86	50	36.76	63.24	61.09
Senior Strategy 1	97	84	13	13.40	86.60	62.83
*χ* ^2^				15.665	15.665	0.145
*P*				<0.001	<0.001	0.704
Senior C-TIRADS	136	86	50	36.76	63.24	61.09
Senior Strategy 2	98	87	11	11.22	88.78	60.81
*χ* ^2^				19.278	19.278	0.004
*P*				<0.001	<0.001	0.953
Junior C-TIRADS	133	80	53	39.85	60.15	63.96
Junior Strategy 1	103	84	19	18.45	81.55	62.67
*χ* ^2^				12.542	12.542	0.081
*P*				<0.001	<0.001	0.776
Junior C-TIRADS	133	80	53	39.85	60.15	63.96
Junior Strategy 2	133	83	50	37.59	62.41	62.61
*χ* ^2^				0.143	0.143	0.087
*P*				0.706	0.706	0.768

Unnecessary biopsy rate = Number of benign nodules in FNA/Total number of FNAs; Positive rate = Number of malignant nodules in FNA/Total number of FNAs; Missed diagnosis rate = (Number of pathologically malignant cases - Number of malignant nodules in FNA)/Number of pathologically malignant cases.

In the junior group, Strategy 1 was associated with a lower unnecessary biopsy rate and a higher positive biopsy rate, again without a statistically significant change in the missed diagnosis rate. In contrast, Strategy 2 did not produce statistically significant changes in unnecessary biopsy rate, positive biopsy rate, or missed diagnosis rate in the junior group (**Table 9**).

## 4 Discussion

Ultrasound remains the first-line imaging modality for thyroid nodule assessment, but interpretation is strongly influenced by reader experience (17, 18). C-TIRADS provides a structured malignancy risk stratification system with practical management recommendations in China. In the present study, C-TIRADS showed good performance in the senior group and lower, but still acceptable, performance in the junior group. These findings support its value as a standardized framework while also illustrating persistent experience-related variability in thyroid ultrasound interpretation.

Importantly, this study was not designed to develop a novel AI algorithm. Rather, we evaluated the clinical use of a commercially available AI-assisted ultrasound system within a C-TIRADS-based workflow. The AI system showed higher sensitivity and accuracy than the junior sonographer group and performance comparable to the senior group, suggesting that it may provide useful adjunctive information, particularly for less-experienced readers. This observation is broadly consistent with previous reports indicating that AI systems can support thyroid nodule classification in ultrasound practice, especially by improving the performance of less-experienced readers while maintaining performance comparable to experienced radiologists (19–21).

Our findings should also be interpreted in the broader context of other ultrasound-based thyroid risk stratification systems. Prior comparative studies have shown that different TI-RADS algorithms may achieve different trade-offs between sensitivity, specificity, and biopsy recommendations. For example, comparative work has suggested that ACR TI-RADS may reduce unnecessary biopsy recommendations compared with some other systems, whereas Korean-style systems tend to favor higher sensitivity at the cost of more biopsies. Similarly, comparisons among the ATA, KTA/KSThR, and ACR systems have shown that differences in category structure and size thresholds can substantially influence both diagnostic performance and management recommendations. In this context, the present findings should be understood as specific to a C-TIRADS-based workflow and should not be assumed to translate directly to other TI-RADS algorithms without further validation (22–24).

The most clinically relevant finding of this study was that the effect of AI assistance differed according to reader experience. In the senior group, flexible reconsideration after reviewing the AI output was associated with better overall performance than mandatory one-level adjustment (**Figure 8**). In contrast, in the junior group, the mandatory adjustment strategy showed clearer benefit than flexible reconsideration (**Figure 9**). One possible explanation is that senior readers may be better able to selectively incorporate AI output while preserving nuanced feature-based judgment, whereas junior readers may benefit more from a predefined rule that reduces subjective inconsistency. However, these findings should be interpreted as preliminary workflow observations rather than definitive practice recommendations.

In this selected cohort, the more favorable strategy for each experience group was also associated with lower unnecessary biopsy rates and higher positive biopsy yields, without a significant increase in missed diagnosis rate (**Table 9**). These biopsy-related findings suggest that AI-assisted workflow integration may have practical value beyond discrimination metrics alone. Because biopsy recommendation is a clinically meaningful downstream consequence of ultrasound interpretation, evaluating these indicators may help contextualize the role of AI in routine practice.

We also found that inconsistent AI results between transverse and longitudinal planes were associated with lower specificity and lower negative predictive value (**Figures 6** and **7**). Nodules smaller than 10 mm and nodules located in the isthmus were more likely to show discordant plane-based AI outputs (**Supplementary Table 3**). These findings suggest that planar inconsistency may identify situations in which AI output should be interpreted more cautiously. They also highlight a potential area for future refinement of commercial AI systems and workflow rules.

Several limitations should be emphasized. First, this was a retrospective single-center study with a relatively limited sample size. Second, because inclusion depended on pathological or cytological verification and complete imaging and AI data, the cohort was enriched for malignancy and does not reflect the prevalence distribution of unselected thyroid nodules in routine practice. This selection pattern may affect estimates of diagnostic performance and limits generalizability. Third, the study assessed group-level consensus performance rather than individual-reader variability. Fourth, all ultrasound acquisition was performed by an experienced operator, which may not fully represent all real-world scanning scenarios. Fifth, the AI system was a commercial proprietary platform, and full details of its internal training dataset and validation framework were not accessible to the investigators. Therefore, the present findings should be interpreted cautiously and require confirmation in larger prospective multicenter studies.

Overall, the present study suggests that integration of a commercial AI tool with C-TIRADS may provide different types of support for sonographers with different levels of experience. Rather than indicating a universal strategy, our results support the need for experience-aware implementation studies when introducing AI into thyroid ultrasound workflow.

## 5 Summary

This retrospective single-center study suggests that a commercial AI-assisted ultrasound system may provide complementary support to C-TIRADS-based thyroid nodule assessment, but the observed benefit appears to depend on reader experience. In this selected pathology-enriched cohort, mandatory one-level adjustment appeared more helpful for junior sonographers, whereas flexible reconsideration appeared more useful for senior sonographers. These patterns were also accompanied by favorable biopsy-related changes without a significant increase in missed diagnoses. However, these findings should be regarded as preliminary workflow observations rather than formal clinical recommendations. Larger prospective multicenter studies are needed before these strategies can be generalized to routine practice.

## Data Availability

The original contributions presented in the study are included in the article/**Supplementary Material**. Further inquiries can be directed to the corresponding authors.
