# Supplementary material for: Experience-dependent integration of a commercial AI system with C-TIRADS for thyroid nodule assessment: a retrospective single-center study
Source: Front Endocrinol (Lausanne). 2026 Aug 3;17:1746242. doi: 10.3389/fendo.2026.1746242 (PMC13477881; doi:10.3389/fendo.2026.1746242)
Supplement: Supplementary file 1 [file Table1.docx]

Table S1. Summary of preoperative FNA cytology and surgical histopathology data used in cohort selection and reference-standard assessment

| Variable | Number of nodules |
| --- | --- |
| Total included nodules | 389 |
| Included nodules with any preoperative FNA cytology data | 280 |
| Included nodules with surgical histopathology as final reference standard | 250 |
| Included malignant nodules confirmed by surgical histopathology | 226 |
| Included benign nodules confirmed by surgical histopathology | 24 |
| Included benign nodules classified by FNA cytology plus stable follow-up | 139 |
| Included nodules with Bethesda I cytology | 0 |
| Included nodules with Bethesda Ⅱ cytology | 139 |
| Included nodules with Bethesda III cytology | 21 |
| Included nodules with Bethesda IV cytology | 44 |
| Included nodules with Bethesda Ⅴ cytology | 42 |
| Included nodules with Bethesda Ⅵ cytology | 34 |
| Included nodules with both preoperative FNA cytology and surgical histopathology | 141 |
| Included nodules with preoperative FNA cytology only | 139 |
| Included nodules with surgical histopathology only | 109 |
| Excluded nodules with inconclusive or unsatisfactory FNA cytology lacking definitive confirmation | 49 |
| Indeterminate cytology nodules analyzed as a dedicated subgroup | 0 |

Note: Bethesda categories refer to preoperative FNA cytology results only. These categories are not mutually exclusive with the surgical histopathology group, because a subset of nodules underwent both preoperative FNA cytology and subsequent surgery. In the present cohort, 141 nodules had both preoperative FNA cytology and surgical histopathology, 139 had preoperative FNA cytology only, and 109 had surgical histopathology only. Final reference-standard assessment was based on surgical histopathology when surgery was performed, or on FNA cytology together with stable follow-up in selected benign nodules. No dedicated subgroup analysis was performed for indeterminate cytology categories.
