# Supplementary material for: Experience-dependent integration of a commercial AI system with C-TIRADS for thyroid nodule assessment: a retrospective single-center study
Source: Front Endocrinol (Lausanne). 2026 Aug 3;17:1746242. doi: 10.3389/fendo.2026.1746242 (PMC13477881; doi:10.3389/fendo.2026.1746242)
Supplement: Supplementary file 2 [file Table2.docx]

|  |  | Sensitivity（%） | Specificity（%） | PPV  （%） | NPV  （%） | Youden Index |
| --- | --- | --- | --- | --- | --- | --- |
| Senior C-TIRADS | 4A | 97.79 | 8.59 | 59.73 | 73.68 | 6.38 |
|  | 4B | 86.28 | 90.80 | 92.86 | 82.68 | 77.08 |
|  | 4C | 51.77 | 97.55 | 96.69 | 59.33 | 49.32 |
|  | 5 | 44.25 | 100.00 | 100.00 | 56.40 | 44.25 |
| Junior C-TIRADS | 4A | 98.23 | 9.20 | 60.00 | 73.68 | 7.43 |
|  | 4B | 78.76 | 82.82 | 86.41 | 73.77 | 61.58 |
|  | 4C | 53.54 | 99.39 | 99.18 | 60.67 | 52.93 |
|  | 5 | 45.13 | 100.00 | 100.00 | 56.79 | 45.13 |

Table S2 Diagnostic Performance of Baseline C-TIRADS at Different Cutoff Points in Senior and Junior Sonographer Groups
