# Supplementary material for: Experience-dependent integration of a commercial AI system with C-TIRADS for thyroid nodule assessment: a retrospective single-center study
Source: Front Endocrinol (Lausanne). 2026 Aug 3;17:1746242. doi: 10.3389/fendo.2026.1746242 (PMC13477881; doi:10.3389/fendo.2026.1746242)
Supplement: Supplementary file 3 [file Table3.docx]

| Table S3 Comparison of Thyroid Nodule Characteristics Between Concordant and Discordant AI Prediction Groups Across Transverse and Longitudinal Planes | | | | | |
| --- | --- | --- | --- | --- | --- |
| Variable | | Concordant AI predictions[n（%）] | Discordant AI predictions [n（%）] | *χ*^2^ | *Ρ* |
| Gender |  |  |  | 1.541 | 0.214 |
|  | Female | 254（84.67） | 46（15.33） |  |  |
|  | Male | 80（89.89） | 9（10.11） |  |  |
| Age (years) | |  |  | 0.583 | 0.445 |
|  | <55 | 232（84.98） | 41（15.02） |  |  |
|  | ≥55 | 102（87.93） | 14（12.07） |  |  |
| Echogenicity |  |  |  | 3.756 | 0.409* |
|  | Hyperechoic | 1（50.00） | 1（50.00） |  |  |
|  | Isoechoic | 62（88.57） | 8（11.43） |  |  |
|  | Hypoechoic | 101（86.32） | 16（13.68） |  |  |
|  | Markedly hypoechoic | 162（84.38） | 30（15.63） |  |  |
|  | Anechoic | 8（100.00） | 0（0.00） |  |  |
| Orientation |  |  |  | 0.565 | 0.452 |
|  | Vertical | 164（87.23） | 24（12.77） |  |  |
|  | Horizontal | 170（84.58） | 31（15.42） |  |  |
| Echogenic foci | |  |  | 3.128 | 0.513* |
|  | Comet-tail artifacts | 1（50.00） | 1（50.00） |  |  |
|  | Punctate echogenic foci of undetermined significance | 152（85.39） | 26（14.61） |  |  |
|  | Macrocalcifications | 41（83.67） | 8（16.33） |  |  |
|  | Peripheral calcifications | 7（87.50） | 1（12.50） |  |  |
|  | No echogenic foci | 133（87.50） | 19（12.50） |  |  |
| Margin |  |  |  | 0.949 | 0.813* |
|  | Circumscribed | 152（87.36） | 22（12.64） |  |  |
|  | Irregular margin | 166（84.26） | 31（15.74） |  |  |
|  | Ill-defined | 6（85.71） | 1（14.29） |  |  |
|  | Extrathyroidal extension | 10（90.91） | 1（9.09） |  |  |
| Nodule diameter | |  |  | 8.351 | 0.004 |
|  | ＜10mm | 173（81.22） | 40（18.78） |  |  |
|  | ≥ 10mm | 161（91.48） | 15（8.52） |  |  |
| Echotexture |  |  |  | 1.568 | 0.211 |
|  | Homogeneous | 164（88.17） | 22（11.83） |  |  |
|  | Heterogeneous | 170（83.74） | 33（16.26） |  |  |
| Halo |  |  |  | 7.406 | 0.109* |
|  | Absent halo | 177（82.71） | 37（17.29） |  |  |
|  | Even a thick halo | 26（100.00） | 0（0.00） |  |  |
|  | Even a thin halo | 40（88.89） | 5（11.11） |  |  |
|  | Uneven thin halo | 28（84.85） | 5（15.15） |  |  |
|  | Uneven thick halo | 63（88.73） | 8（11.27） |  |  |
| Location |  |  |  | 9.292 | 0.026 |
|  | Isthmus | 22（68.75） | 10（31.25） |  |  |
|  | Upper | 173（87.37） | 25（12.63） |  |  |
|  | Lower | 67（84.81） | 12（15.19） |  |  |
|  | Middle | 72（90.00） | 8（10.00） |  |  |

Note: *Calculated using Fisher's exact probability method
